# Identifying postpartum intervention approaches to prevent type 2 diabetes in women with a history of gestational diabetes

**DOI:** 10.1186/1471-2393-11-23

**Published:** 2011-03-24

**Authors:** Jacinda M Nicklas, Chloe A Zera, Ellen W Seely, Zainab S Abdul-Rahim, Noelle D Rudloff, Sue E Levkoff

**Affiliations:** 1 Division for Research and Education in Complementary and Integrative Medical Therapies, Harvard Medical School, Boston, MA, USA; 2 Department of Endocrinology, Diabetes and Hypertension, Brigham and Women's Hospital, Harvard Medical School, Boston, MA, USA; 3 Division of Maternal-Fetal Medicine, Department of Obstetrics and Gynecology, Brigham and Women's Hospital, Boston, MA, USA; 4 Division of Women's Health, Department of Medicine, Brigham Women's Hospital, Boston, MA, USA; 5 Department of Psychiatry, Brigham and Women Hospital, Boston, MA, USA

## Abstract

**Background:**

Women who develop gestational diabetes mellitus (GDM) have an increased risk for the development of type 2 diabetes. Despite this "window of opportunity," few intervention studies have targeted postpartum women with a history of GDM. We sought perspectives of women with a history of GDM to identify a) barriers and facilitators to healthy lifestyle changes postpartum, and b) specific intervention approaches that would facilitate participation in a postpartum lifestyle intervention program.

**Methods:**

We used mixed methods to gather data from women with a prior history of GDM, including focus groups and informant interviews. Analysis of focus groups relied on grounded theory and used open-coding to categorize data by themes, while frequency distributions were used for the informant interviews.

**Results:**

Of 38 women eligible to participate in focus groups, only ten women were able to accommodate their schedules to attend a focus group and 15 completed informant interviews by phone. We analyzed data from 25 women (mean age 35, mean pre-pregnancy BMI 28, 52% Caucasian, 20% African American, 12% Asian, 8% American Indian, 8% refused to specify). Themes from the focus groups included concern about developing type 2 diabetes, barriers to changing diet, and barriers to increasing physical activity. In one focus group, women expressed frustration about feeling judged by their physicians during their GDM pregnancy. Cited barriers to lifestyle change were identified from both methods, and included time and financial constraints, childcare duties, lack of motivation, fatigue, and obstacles at work. Informants suggested facilitators for lifestyle change, including nutrition education, accountability, exercise partners/groups, access to gyms with childcare, and home exercise equipment. All focus group and informant interview participants reported access to the internet, and the majority expressed interest in an intervention program delivered primarily via the internet that would include the opportunity to work with a lifestyle coach.

**Conclusion:**

Time constraints were a major barrier. Our findings suggest that an internet-based lifestyle intervention program should be tested as a novel approach to prevent type 2 diabetes in postpartum women with a history of GDM.

**Trial Registration:**

ClinicalTrials.gov: NCT01102530

## Background

Gestational diabetes mellitus (GDM), defined as glucose intolerance that begins or is first detected during pregnancy [1], occurs in 3-5% of pregnancies in the United States (US), [2] and carries a 7-12 fold lifetime risk for the subsequent development of type 2 diabetes [3]. Type 2 diabetes results in substantial morbidity, including cardiovascular, renal, and retinal complications, and is the fifth leading cause of death in the US. Data from several regions of the US suggest increases in GDM incidence over the past decade [4-8]. An intervention targeting postpartum women with a history of GDM thus offers a unique opportunity both for the primary prevention of type 2 diabetes and for the reduction of individual, as well as societal, disease burden. Furthermore, evidence from post-hoc subgroup analyses from the Diabetes Prevention Program demonstrated that an intensive behavioral lifestyle intervention to prevent type 2 diabetes was equally effective among women with and without self-reported prior history of GDM [9].

Despite this "window of opportunity" to prevent type 2 diabetes, and the promise of lifestyle interventions to reduce the risk of type 2 diabetes in women with a history of GDM [10], few lifestyle intervention studies have focused on this at-risk population. Lifestyle intervention trials targeting weight loss in the general postpartum population have numerous methodological limitations, including small sample sizes [11,12], lack of data on refusal rates [11,12], high attrition rates [11,12], lack of clarity as to the use of intent to treat analyses [13], poor adherence [14] and lack of efficacy [14]. In one survey of women with a history of GDM, although 90% knew that GDM was a risk factor, only 16% perceived themselves to be at high risk for type 2 diabetes [15]. In addition, several studies demonstrate that women with a history of GDM are no more likely to adopt healthy lifestyle behaviors like diet and exercise than women with no history of GDM [16-18]. Women with a history of GDM who would like to modify their risk for type 2 diabetes describe multiple barriers to lifestyle change, including lack of time and energy due to competing work and family demands, and difficulties obtaining child care [19-21].

This study builds on the existing literature in seeking the perspectives of women with a history of GDM to identify barriers and facilitators to the adoption of, and adherence to, healthy lifestyle changes in the postpartum period. In addition, this study seeks to identify specific intervention approaches that would facilitate the participation of postpartum women with a history of GDM in a lifestyle intervention program.

## Methods

### Sample

We recruited prospective participants through flyers and internet postings. Women were eligible if they reported having had a history of GDM within the previous seven years, were between 18 and 50 years old, were able to communicate in English, and reported that they had never been told that they had type 2 diabetes.

### Study Design

Although we had planned on conducting in-person focus groups exclusively, the scheduling difficulties for study participants made it clear that it would be impossible to conduct an adequate number of focus groups to achieve data saturation within a reasonable timeframe. Rather than limit our sample to those individuals who were able to attend a focus group, we employed a mixed methods approach, combining focus groups with informant interviews. Women were first asked to attend a focus group and offered three possible dates and times for study participation. Those who could not attend any one of the offered times were then invited to participate in an informant interview by telephone.

Of the 43 women who contacted the project staff and expressed interest in participating in a focus group, four were not eligible (three had GDM >7 years prior; one currently had type 2 diabetes), and one declined after hearing about the study. Of the remaining 38 women eligible for study participation, all expressed interest in participating in a focus group, but only ten were able to accommodate their schedules to attend one of the three possible focus groups offered. Two focus groups were held, including three and seven participants, respectively.

Of the 28 remaining women interested in, but unable to attend a focus group, 19 women participated in informant interviews, with 18 participants completing the whole interview. Of the 18 informants with complete data, 3 were removed from the study sample because they were in their first GDM pregnancy, and thus could not comment on the experience of being postpartum following a pregnancy complicated by GDM. Thus we present data from 10 focus group participants and 15 complete informant interviews for this analysis, for a total of 25 participants.

#### Focus groups

Focus groups were led by a trained facilitator, while a research assistant took field notes. Written informed consent was obtained at the beginning of the focus group. Participants were asked to recall their most recent postpartum year following a pregnancy complicated by GDM. An interview guide outlined domains of interest, but allowed the facilitator latitude to explore other themes that emerged during the focus groups (Table 1). The focus groups lasted 70 minutes on average and were digitally recorded and transcribed for analysis. Focus group participants received compensation for childcare and transportation costs. Focus groups were conducted May-June 2009.

**Table 1:** Domains of interest and themes covered in focus groups and informant interviews

Focus Groups and Informant Interviews	Informant Interviews Only
Participant knowledge and attitudes about postpartum diabetes screening	Perceived risk for type 2 diabetes

Barriers to healthy eating and physical activity in the year after delivery	Facilitators to healthy eating and physical activity in the year after delivery

Interest in participating in a lifestyle intervention in the year after delivery	Importance of social supports being involved in a lifestyle intervention program

Preferred design of a lifestyle intervention program for the year after delivery	

Preference to work with a lifestyle coach* either a) one-on-one, b) in a group, or c) some combination of individual and group coaching	

* The lifestyle coach was defined as someone to "help you stay on track, motivate you, answer your questions and help you to achieve your goals."

#### Informant interviews

Informant interviews were conducted by telephone by the same research assistant who took field notes during the focus groups. Informed consent was obtained at the onset of the interview and informant interviews followed a semi-structured script, including both closed and open-ended questions. If participants did not respond to open-ended questions, the research assistant would probe systematically for answers. The informant interviews allowed us to cover topics that were included as domains of interest for the focus groups, but were not discussed because of the natural flow of focus group conversation within the time constraints of a focus group session (Table 1). Similar to the focus groups, informant interview participants were asked to recall their experiences during their first year postpartum subsequent to their most recent pregnancy complicated by GDM. Telephone interviews lasted from 20-45 minutes; the research assistant took notes. Participants who completed telephone interviews received a gift card as compensation. Informant interviews were conducted March-December 2009.

The Institutional Review Boards at Brigham and Women's Hospital and Harvard Medical School approved both the focus groups and informant interviews. Participants in the focus groups provided written consent and participants in informant interviews provided verbal consent of their participation.

### Data Analysis

Using grounded theory, two researchers independently employed open-coding to identify themes from focus group transcripts and field notes. The researchers then came to consensus on representative data and a final set of themes [22]. For the informant interviews, data analysis consisted of frequency distributions.

## Results

The mean age of the 25 study participants was 35 years (range 26-42); with a mean pre-pregnancy BMI of 28 (range 15-50). All had experienced at least one prior GDM pregnancy, with five having had two, and one having had three prior pregnancies complicated by GDM. Five women were pregnant with GDM at the time of the interview. Women were an average of 1.7 years (range 2 months - 7 years) from the delivery of their last GDM pregnancy (Table 2). Fifty-two percent of participants were Caucasian, 20% were African-American, 12% were Asian, and 8% were American Indian, and 8% refused to specify. Participants had a mean number of 1.9 children (SD 0.4) with a mean age of 3.7 (SD 2.8).

**Table 2:** Participant characteristics for focus groups and informant interviews among women with a history of gestational diabetes (n = 29)

Characteristic	Focus group (n = 10)	Informant interview (n = 15)	Total Sample (n = 25)
Age, mean (SD)	36 (6.0)	35 (4)	35 (5)

Race:			
White, N (%)	4 (40%)	9 (60%)	13 (52%)
African American, N (%)	5 (50%)	0 (0%)	5 (20%)
Asian, N (%)	1 (10%)	2 (13%)	3 (12%)
American Indian, N (%)	0 (0%)	2 (13%)	2 (8%)
Refused	0 (0%)	2 (13%)	2 (8%)

Pre-pregnancy BMI, kg/m^2^, mean (SD)	31 (11)	26 (5)	28 (8)

Number of GDM pregnancies, mean (SD)	1.1 (0.3)	1.8 (0.7)	1.4 (0.6)

Time since last GDM pregnancy, years, mean (SD)	1.8 (2.1)	1.3 (0.8)	1.7 (1.7)

Currently pregnant, N (%)	1 (10%)	4 (27%)	5 (20%)

Number of children, mean (SD)	1.9 (0.3)	2.0 (0.5)	1.9 (0.4)

Mean age of children, mean (SD)	4.9 (3.5)	2.7 (1.6)	3.7 (2.8)

### Perceived risk for type 2 diabetes

#### Informant interviews

10/15 informants felt they were at moderate or high risk for type 2 diabetes over the next 10 years, whereas 5/15 felt they had a slight or almost no chance of developing type 2 diabetes. One woman explained that she felt that she had almost no chance because she had a history of three GDM pregnancies and had not changed her lifestyle and "has been fine."

#### Focus Groups

Focus group participants expressed their concern about developing type 2 diabetes in the future, especially if they did not change their lifestyles. One participant stated, "I don't want to have regular diabetes if I don't take care of myself." Another said she was, "very concerned about not having lost the weight."

### Barriers to healthy eating postpartum

#### Focus groups

Barriers to adopting healthy eating behaviors postpartum fell into several categories, including difficulty shopping with children, child food preferences, time and financial constraints, and obstacles at work (Table 3).

**Table 3:** Reported barriers to healthy eating postpartum among focus group participants with a history of gestational diabetes

Theme	Representative Quotes
**Constraints related to children**	"Confusing nutrition labels in store and with kids pulling on you, there is no time to read labels." "When I make healthy meals, my kids won't eat it--they want fast food." "My kids always ask for and get the snacks/junk--cookies, fried foods, so when I try to provide healthy food, they object."

**Time constraints**	"I need more time to go to the grocery store and get better at planning out in advance healthy meals and snacks." "Impossible to have the whole family sit down and eat well with schedules" "I would [in the past] count my calories that I took in during the day and I would go to the gym and work it off--I just don't have the time to do that with kids."

**Financial constraints**	"Cost of smart shopping is too high." "Going to get a salad at the salad bar costs more than a dollar cheeseburger off the value menu"

**Difficulty at work**	"Sweets are difficult because they are often available at work. Meetings have danishes and muffins, cheese plates." "Vending machines at work never have healthy options."

#### Informant interviews

Almost half of the women who participated in the informant interviews (7/15) said that they found it difficult to maintain a healthy diet during the first 12 months postpartum. Many discussed similar barriers to those expressed by the focus group participants, stating they were busy with their children and did not have time to eat a healthy diet. Several informants felt that going back to work was a major barrier. One woman said that she ate whatever her children were eating because she was working full-time and did not have time to prepare separate meals. Another woman commented that she ate a lot of unhealthy fast food and frozen food because of her work schedule. One informant said, "Working full-time is a year-round obstacle." Another informant attributed her difficulty with healthy eating to nursing: "In the beginning when I was nursing all the time, I was just starving. So I would just eat tons of food." In addition, one informant said she was able to make better choices when she was less sleep deprived, stating "as I got more sleep, it was easier to think about preparing healthy meals instead of just eating whatever I could find." Another informant discussed her financial barrier to healthy eating, stating "good food is expensive."

### Facilitators to healthy eating postpartum

#### Informant interviews

When asked what helped or would have helped to facilitate healthy eating postpartum, 4/15 informants mentioned meal planning, including sample menus. Four informants mentioned nutrition education, including lists of healthy foods to add to the diet, grocery shopping tutorials, learning ways to prepare healthy foods quickly, working with a nutritionist, and learning about portion sizes. One informant wished she had access to healthy food already prepared and frozen; another wanted vouchers to help her to afford healthy foods. Four informants said they continued to rely on all or some components of the GDM diet. Three informants felt that increased accountability, either to a group, to a physician doing weight checks and/or blood tests, or by logging in personal data on-line, would have helped them. One informant suggested that including her children in meal preparation would help them accept dietary changes.

### Barriers to physical activity postpartum

#### Focus groups

The major themes identified as barriers to postpartum physical activity included lack of motivation, time constraints, and financial constraints (Table 4).

**Table 4:** Reported barriers to physical activity among focus group participants with a history of gestational diabetes

Theme	Representative Quotes
**Lack of motivation/Fatigue**	"Getting up and off the couch and motivated." "Tired both physically and mentally."

**Time constraints related to children**	"Kids schedules, carting kids around to their sports events, childcare."

**Time constraints**	"No time to fit exercise into your daily hectic life." "I have a gym in my building and I still don't have the time to exercise!"

**Financial constraints**	"Joining a gym is expensive."

#### Informant interviews

The majority of informants (11/15) said they had difficulty exercising in the first year postpartum. Similar to the focus groups, fatigue (4/15) and lack of time (5/15) were most commonly cited. One woman stated, "[my] kids are too young. It is not about you but the kids. [Feels] like a hostage situation; I keep getting fatter and fatter." Another said she planned to start exercising in a year, when her children would be in school. Three informants said going back to work made it more difficult to find time to exercise. One said, "I couldn't find the time when I went back to work - [I was] discouraged by gaining weight back." Guilt was also identified as a barrier to exercising by the informants. One informant said, "I was exhausted and already feeling so guilty for being away from my child while I was working, so I did not exercise."

### Facilitators to physical activity postpartum

#### Informant interviews

When asked about factors that would facilitate postpartum physical activity, 12/15 informants completing this portion of the interview said finding an exercise buddy or a group (including group exercise classes). Seven women said they exercised with their babies, including walking with a stroller or baby carrier. One informant said she tried to "get creative," by holding her baby and doing squats and other exercises at home. Nine informants said that a gym membership, especially with childcare included, would facilitate physical activity, but several said they could not afford memberships. Three women indicated that having home exercise equipment facilitated or would facilitate physical activity, and three used home exercise DVDs. Three said the cold weather made it difficult to exercise in winter; they found it easier to exercise in summer. Two informants said they tried to integrate exercise into daily life; one said she always walked upstairs to change her baby's diaper and one always used the stairs at work.

### Preferred design of a postpartum intervention program

#### Focus groups

All focus group participants expressed interest in a postpartum intervention program but acknowledged that participation would be difficult given their time constraints. One woman said she had looked for a program but was unable to find one. As participants in the larger focus group responded to how they would want an intervention delivered, they began to discuss working with clinicians during their GDM pregnancies. Their experience feeling judged by medical doctors emerged as a major theme. One woman said, "not that they [clinicians] mean to be, but they are very clinical and removed and don't seem to understand, you are attempting to deliver a healthy baby and manage and plan the rest of your life; whereas they are with you for 20 minutes and are attempting to determine why you decided to eat your toast with jam!" Another woman stated, "I found myself very annoyed at the clinicians because I always felt they were a tinge judgmental about the GDM and had a lot of assumptions. Any meeting with them started with, 'now you have to change your lifestyle,' and I thought, you don't know what my lifestyle is, so how do you know what is bad or what needs to change. I may already know and be changing what I need to in order to be healthy. I am not a child." Participants in this focus group came to a consensus that, "when doctors tell you what to do without understanding your daily life, it is not helpful."

The majority of the focus group participants felt that, considering their time constraints, an internet-delivered intervention would be more feasible than meeting exclusively in groups, and many suggested a combination of an internet-based intervention along with group meetings. None of the participants felt that a primarily telephone-delivered intervention would work for them (Table 5). There was unanimous agreement and enthusiasm about the idea of working with a "lifestyle coach," described by the facilitator as someone to "help you stay on track, motivate you, answer your questions and help you to achieve your goals."

**Table 5:** Preferred design of postpartum intervention program among focus group participants with a history of gestational diabetes

Theme	Representative Quotes
**Focus of intervention**	"GDM comes with a whole team of professionals, but what is missing is a place to bounce off how to move forward [after delivery] with life ideas in a positive surrounding, as opposed to looking back at mistakes."

**Interest in group meetings**	"Group meetings [are] good for socialization and social support." "Helpful to hear from peers, but so busy/impossible to schedule." "Start out with a group meeting so everyone gets to know each other and let them decide what they want the interactions to be, [like] maybe meeting again on a regular basis, then contact online. If you don't get to know anyone by seeing them in person first, you won't feel connected to an online group." "If time were not an issue, getting out of the house to a gym and/or organized exercise class would be fun and ideal." "Timing is key, we don't have very much of it!"

**Interest in an internet-based intervention**	"Use of internet convenient" "Not just internet alone."

**Interest in an phone-based intervention**	"Phones are too much of a hassle to hold! "

**Interest in a lifestyle coach**	"I like the idea of a lifestyle coach because it seems more like a partner than someone who will talk down to you. With a coach you are a client, whereas with a doctor you are a patient."

#### Informant interviews

All participants (15/15) expressed interest in a lifestyle intervention program and working with a lifestyle coach. Four preferred one-on-one interactions with a lifestyle coach, and most (11/15) preferred a combination of lifestyle coach and group.

Almost all study informants (13/15) said they would be interested in an internet-based program. One said, "internet is more convenient because the times are flexible." Another stated, "Have a peer group in-person to start, to get to know each other, then use chat rooms/email to access at all times of the night." Another specifically indicated that she would not want to use the phone, "primarily for convenience. I like being able to re-reference information." Among those two informants who would not choose an internet-based intervention, one said she was "not interested in internet program because I am a people person and would prefer face-to-face time weekly." Only 2/15 would choose a phone-based intervention. One informant said, "If someone caught me at the right time [I] would prefer phone."

### Regular use of and comfort level with the internet

#### Focus groups

All focus group participants were "very comfortable" using the internet, and nearly all used the internet daily.

#### Informant interviews

All informants (15/15) rated themselves as "very comfortable" using the internet, and all said they used the internet daily.

### Importance of social supports being involved in a lifestyle intervention program

#### Informant interviews

12/15 informants wanted other people included in the intervention program to provide social support for healthy behavioral changes. Of these, eleven women wanted their spouse or partner included in the program. One informant said she would, "need [my] husband to support dietary changes," and another wanted her "husband aware of [my] commitment." Another informant wanted her spouse involved "...so that we can make time for each other to work out." Six informants said they would want the entire family/household included in an intervention. One said she would need "family on board because I can't make two separate meals." Another said, "awareness is probably the biggest thing [to help them be] supportive of your goals." One informant wanted to "explain to the family the continued need for diet and exercise modification after delivery. They think the diabetes goes away... the whole family could benefit from modified lifestyle."

## Discussion

In this study, discussions with women with a history of GDM through focus groups and informant interviews provide information about barriers and facilitators to healthy lifestyle choices in the year postpartum, and they discuss their preferred design and components of a lifestyle intervention program. Women in this population found it difficult to attend a single focus group, and most stated that they would have difficulty with an intervention based exclusively on in-person group sessions or contacts by phone. This evidence of significant time constraints suggests that implementing a face-to-face lifestyle intervention like the Diabetes Prevention Program [9] would be difficult in this population. An intervention that does not require face-to-face contact that women can utilize at their convenience may have more success. In our study, the majority of participants expressed interest in an internet-based lifestyle intervention that they could access on their own schedules. We found universal familiarity and high levels of daily internet use in this population, similar to rates found in a recent survey of this age group [23]. This is a novel and important finding, as the cost of internet-based technologies has decreased significantly, making it more feasible and potentially cost-effective to implement internet-based interventions. Given the high risk for type 2 diabetes in the GDM population and the need for effective intervention strategies in the postpartum "window of opportunity," these data provide valuable information for the design of postpartum interventions with the goal of decreasing the incidence of type 2 diabetes.

A larger proportion of the women in our study considered themselves to be at moderate or high risk for type 2 diabetes than has been seen in previous studies [15,24]. This may be due to the fact that the study took place at an academic medical center and may have included more women exposed to GDM education. Despite this higher level of perceived risk, participants nonetheless reported difficulties adopting healthy lifestyle changes, citing barriers similar to those found in other studies [24-26]. Similarly, Swan et. al. demonstrated that rural Australian women with a history of GDM had a low prevalence of healthy diet and physical activity behaviors despite increased perception of risk and knowledge about prevention strategies for type 2 diabetes [21]. Previous studies identified lack of time and lack of childcare as the most important barriers to healthy lifestyle activities in women with prior GDM [25], findings which are echoed in our study. In addition, women in our study discussed how financial constraints, difficulties related to work, and feeling guilty about being away from their children all served as barriers to achieving a healthy lifestyle.

A potentially important novel finding was the frustration expressed by participants with feeling judged by clinicians about their lifestyle and choices, which may be important to address in this population. Respondents' reports of feeling judged by their health care providers may signify untoward effects of the "medical model," which assumes that if individuals are provided adequate information and skills and are motivated to change their behaviors, they will naturally make informed choices and modify their unhealthy behaviors [27]. It is possible that more collaborative approaches to behavior change such as patient-centered counseling or motivational interviewing techniques, may be more acceptable in this population, [28] and that counseling delivered by non-clinicians may feel less judgmental and perhaps could be more successful. These findings should be considered when designing future intervention studies in this population. In our study, we found that women were enthusiastic about the idea of a lifestyle coach, whom one woman stated would seem "more like a partner."

We identified several key concepts that may be useful to integrate into future postpartum interventions for women with a history of GDM. We found that only ten of 38 potential participants who indicated their desire to attend a one-time focus group were able to make arrangements to attend, and nine of these 38 women could not be scheduled for an informant interview. This is consistent with the findings from a recent postpartum weight loss trial for overweight and obese women in which participants were enthusiastic about group meetings and exercise sessions, but on average attended only 3.8 classes out of the eighteen that were specified for the protocol, and 43% did not attend a single session [14]. Given the difficulty with retention seen in previous studies employing face-to-face interventions, as well as the time constraints identified in our study, an intervention that women can utilize at their convenience may have more success. We identified substantial interest in an internet-based intervention, with some women expressing interest in face-to-face interactions to complement the internet-based format.

Both focus group participants and informants discussed financial barriers to the adoption of healthy lifestyle changes, including the high cost of healthy foods and gym memberships. In addition, many participants discussed difficulties convincing their children to eat healthy foods, and the majority of informants wanted their spouses/partners or other members of their family included in an intervention program. Informants discussed the importance of family awareness and support, and also mentioned that lifestyle changes would benefit the entire family. It has become increasingly clear that public health interventions that focus specifically on individual-level determinants (e.g., attitudes, beliefs, and skills) are less successful than those that take into account the social ecological contexts in which the interventions occur [29]. Including an ecological perspective to design a postpartum intervention program would incorporate multiple levels of influence on health, including individual, interpersonal/familial, community, and public health.

### Limitations

This study is limited by its small sample size, and the fact that it was only conducted in English; therefore these women may not be representative of the general population of women with a history of GDM. Since we were asking women to recall barriers and facilitators in the postpartum year, the responses may be affected by recall bias given the varying amount of time since their last GDM pregnancy. In addition, some of the participants may have given answers they thought we wanted to hear, reflecting a social desirability bias [30]. Finally, the quality of the informant interviews may have varied given that many women were multi-tasking while participating in the interview, including caring for their children or driving home from work.

## Conclusions

Findings from this study may be useful to aid in the development of lifestyle intervention programs for postpartum women with a history of GDM. Based upon our study, a lifestyle intervention program that is primarily internet-based that includes a lifestyle coach, integrates spouses/partners and other family members, and includes strategies to address barriers including limited time, limited finances, and childcare demands, may be feasible and acceptable for postpartum women with a history of GDM. Alternatively, given the time constraints and other barriers to lifestyle changes found in this study, future studies may want to investigate other non-lifestyle based interventions, including pharmacologic prevention of type 2 diabetes.

## List of abbreviations

GDM: gestational diabetes mellitus

## Competing interests

The authors declare that they have no competing interests.

## Authors' contributions

JN contributed to the design, implementation, analysis and interpretation of results, wrote and edited the manuscript. CZ contributed to the design and edited the manuscript. ES conceived the study, contributed to the design, implementation, monitoring and conduct of the study and analysis, and edited the manuscript. ZA contributed to the design, scheduled participants, took field notes, collected and entered data, and edited the manuscript. NR contributed to the design, conducted focus groups, transcribed the data, and conducted the analysis. SEL conceived the study, contributed to the design, implementation, analysis and interpretation of results, wrote and edited the manuscript. All authors approved the final draft of the manuscript.

## Pre-publication history

The pre-publication history for this paper can be accessed here:

http://www.biomedcentral.com/1471-2393/11/23/prepub
